# Facilitation influences patterns of perennial species abundance and richness in a subtropical dune system

**DOI:** 10.1093/aobpla/ply017

**Published:** 2018-03-23

**Authors:** Cecilia E S Dalotto, Rafael B Sühs, Michele S Dechoum, Francisco I Pugnaire, Nivaldo Peroni, Tânia T Castellani

**Affiliations:** 1 Programa de pós-graduação em Ecologia, Universidade Federal de Santa Catarina, CEP, Florianópolis, Santa Catarina, Brazil; 2 Programa de pós-graduação em Biologia de Fungos, Algas e Plantas, Universidade Federal de Santa Catarina, CEP, Florianópolis, Santa Catarina, Brazil; 3 Estación Experimental de Zonas Áridas, EEZA-CSIC, Ctra. de Sacramento s/n, La Cañada, Almería, Spain

**Keywords:** *Guapira opposita*, perch effect, plant community, plant–plant interactions, positive interactions, succession, vegetation dynamics

## Abstract

Positive interactions in plant communities are under-reported in subtropical systems most likely because they are not identified as stressful environments. However, environmental factors or disturbance can limit plant growth in any system and lead to stressful conditions. For instance, salinity and low nutrient and water availability generate a gradient of stressful conditions in coastal systems depending on distance to shore. In a tropical coastal system in SE Brazil, we aimed to assess whether *Guapira opposita*, a shrub common in *restinga* environments, acted as nurse involved in ecological succession and which factors influenced its facilitation process. We sampled perennial species above 10 cm in height under the canopy of 35 *G. opposita* individuals and in neighbouring open areas. Shrub height, canopy area and distance to freshwater bodies were measured in the field, and distance to the ocean was obtained from aerial images. In addition, we measured the distance to the closest forest patch as a potential source of seeds. Plant abundance and species richness were higher under the canopy of *G. opposita* than in open areas. Facilitation by *G. opposita* was mainly determined by shrub height, which had a positive relationship with woody and bromeliads abundance and species richness while there was no relationship with the other factors. Overall, our data evidence that tropical environments may be highly stressful for plants and that nurse species play a key role in the regeneration of *restinga* environments, where their presence is critical to maintain ecosystem diversity and function.

## Teaser

We assessed whether *Guapira opposita*, a common shrub in coastal sand dunes in southern Brazil, was involved in ecological succession and which factors influenced its positive effects (facilitation) on other plant species. Woody plants and bromeliad abundance and richness were higher under the canopy of *G. opposita* than in open areas. Facilitation was mainly determined by the height of *G. opposita* individuals, and was not influenced by shrub area, distance to freshwater bodies, to the sea or to forest patches. Our data evidence that facilitator species play a key role in *restinga* regeneration and their presence is critical to maintain ecosystem diversity.

## Introduction

The process of ecological succession refers to directional changes in the composition of species and community structure over time (Clements 1916). In a successional process guided by facilitation, some species are central to the establishment of others by improving or mitigating conditions, which allows less tolerant species to establish (Connell and Slatyer 1977). This model of succession, centred on focal individuals, is known as nucleation (Yarranton and Morrison 1974). From these nuclei, some shrub or tree species can recruit different individuals under their canopies and expand to form large patches of continuous vegetation, affecting the composition of future forests (Yarranton and Morrison 1974; Slocum 2001).

Shrubs and trees that benefit other plant individuals below their canopy, in both early or late successional stages, are called nurse or facilitator species (Niering *et al.* 1963). The benefit provided by these species occurs through the mitigation of environmental conditions and changes in distribution of resource availability, which can affect local species richness and plant abundance and alter the regeneration dynamic of the whole community (McIntire and Fajardo 2014). In environments under high abiotic stress, such as coastal systems, nurse species allow less stress-tolerant species to become established, increasing local abundance and richness (Armas and Pugnaire 2009; Cavieres *et al.* 2014).

Some specific plant characteristics can improve the facilitation–nucleation process. For instance, species with large crowns and dense canopies are often good facilitators (Arantes *et al.* 2014), because they reduce incident radiation and soil temperature, as well as promote an increase in nutrient availability (Callaway 2007; Brooker *et al.* 2008). In addition, species with fleshy fruits attract seed dispersers and act as perches, increasing seed rain under their canopies (McConkey *et al.* 2012). Thus, shrubs with these traits can improve local conditions and receive propagules *via* the attraction of animal dispersers, increasing seedling recruitment under their canopy (Slocum 2001). In the potential nursing effect, distance to seed sources is an important factor as dispersal (hence seed availability) tends to decrease with distance (Martínez-Garza *et al.* 2009). Therefore, the shorter the distance to seed sources, the greater the number of propagules arriving.

The main factors that influence facilitation under shrubs in coastal ecosystems are linked to environmental stress, such as temperature, wind and salinity, or the scarcity of resources, such as lack of water or nutrients (Maestre *et al.* 2009). In stressful environments, the frequency of facilitative interactions is expected to predominate over competitive interactions (Bertness and Callaway 1994; Callaway 2007). Thus, stressful coastal environments offer good conditions to assess facilitation processes, since salinity and low soil water availability create gradients of stress that tend to decrease away from the ocean and near to freshwater bodies, respectively. These two gradients may have different directions and at times cancel each other, but individually they could potentially match the stress-gradient hypothesis (SGH; Bertness and Callaway 1994) because, with the reduction of physical severity, facilitative interactions are expected to become less important than competitive interactions in structuring plant communities (Bertness and Callaway 1994; Pugnaire and Luque 2001; Callaway 2007; Callaway and Pugnaire 2007).

Brazilian *restinga* environments comprise a diverse group of vegetation types along the Brazilian coast that belong to the Atlantic Forest biome. These vegetation types have developed on sandy, poor soils of marine, alluvial or wind origin. The plant communities are diverse and dominated by stress-tolerant species that often provide adequate conditions for the germination and growth of less stress-tolerant species (Scarano 2002). Environmental severity in *restingas* is linked to nutrient limitation, high radiation, low soil water availability, strong winds and burial by sand (Shumway 2000). Early colonizer species often act as facilitators, with an important role in ecological succession and in the transition from an herbaceous to an arboreal physiognomy (Castanho *et al.* 2015). Therefore, it could be expected that in *restinga* environments woody species establishment would be higher under the canopy of stress-tolerant shrubs than in the herbaceous vegetation matrix or in bare sand. In southern Brazil, *Guapira opposita* (Nyctaginaceae) is a common species in coastal dune systems (Reitz 1970). It is considered a nurse species (Castanho *et al.* 2012), but its role in community composition and dynamics has not been assessed.

Information on facilitation in tropical and subtropical environments is still limited, despite its importance to understand how succession occurs in *restingas*. Thus, our main objective was to understand the role of *G. opposita* in a *restinga* environment, and specifically (i) evaluate whether richness and abundance of woody species under the canopy of *G. opposita* differed from areas dominated by herbaceous vegetation; i.e., its facilitation effect; (ii) assess how *G. opposita* morphology, distance to the ocean and freshwater bodies influence facilitation; and (iii) evaluate the influence of distance to forest patches on the facilitation effect. We hypothesized that (i) abundance and richness of woody species will be higher under *G. opposita* shrubs than in surrounding areas dominated by herbaceous vegetation; (ii) facilitation will be more intense under larger and taller *G. opposita* individuals than under smaller individuals; (iii) facilitation will depend on environmental stress, which increases with distance to freshwater bodies and with proximity to the ocean, in parallel to gradients of water availability and salinity; and (iv) proximity to forest patches will positively influence plant establishment under the canopy of *G. opposita*.

## Methods

### Study system

The study was conducted in Dunas da Lagoa da Conceição Municipal Park (DLCMP), a dune system on the eastern coast of Santa Catarina Island, Florianópolis, Brazil (27°37′33″S – 27°37′53″S, 48°27′28″W – 48°27′08″W). The climate type is *Cfa* (mesothermal moist) according to the Köppen–Geiger climate classification. Mean temperature ranges from 26 °C in summer to 16 °C in winter, with an annual mean of 20 °C (INMET 2017). Rainfall is well distributed throughout the year but is more intense during the summer months, with an annual average of 1500 mm (INMET 2017). Interspersed within the moving and semi-fixed sand dunes there are permanent freshwater bodies in dune slacks. These freshwater bodies are temporarily flooded in rainy months (in the summer) by groundwater that emerges naturally from below the surface (Beduschi and Castellani 2013). The plant community is typical for *restinga* and ranges from herbaceous to shrubby physiognomy, including larger shrubs in vegetation bands further from the ocean. Most of the system is dominated by an herbaceous-subshrub matrix with either isolated or densely clustered shrubs (Guimarães 2006).

*Guapira opposita* is a widely distributed species in Brazil, where it occurs in the Atlantic Forest, Amazonian, Caatinga and Cerrado domains (Patrícia *et al.* 2000). In the south-east, its distribution is restricted to rainforests along the Atlantic coast and *restingas*. The species is highly polymorphic (e.g. it can be 1 to 25 m tall), which is directly influenced by the environment where it occurs. In shallow or rocky soils it assumes a dwarf size; in *restinga* environments its leaves are slightly shorter and more coriaceous than in the rain forest. Ripe fruits are fleshy and red to purple, and attract birds that act as dispersers. It may be abundant in *restingas*, sometimes forming dense thickets on the sandy, slightly undulating terrain near the beach (Reitz 1970).

The sampling area comprises a section of dunes inside the DLCMP, between 50 and 600 m from the ocean. Individuals of *G. opposita* are common in the area as isolated individuals or in small nuclei with individuals of *Clusia criuva* (Clusiaceae), *Myrcia palustris* (Myrtaceae) and *Ocotea pulchella* (Lauraceae). Nuclei vary in size (1.5–5 m^2^) and increase over time (C. E. S. Dalotto, pers. comm.). Forest patches close to arboreal *restingas* sharing similar plant species can also be found. These patches are very similar in size and occupy 2 ha on average. Shallow, subjected to temporary flood are common, especially in periods of intense rain.

### Sample design

Thirty-five *G. opposita* shrubs, at least 20 m apart from each other, were randomly selected and georeferenced. For each shrub, we measured canopy diameter to calculate the area as that of an ellipse, as well as height and distance to the closest freshwater body. Under the canopy of each shrub, we sampled all individuals of woody species with a minimum height of 10 cm, as well all individuals of bromeliad species. Plants were identified to the species level or, when not possible, to the genus level.

We randomly selected an area similar in size and near to every sampled shrub (open areas) and all bromeliads and woody individuals at least 10 cm tall were sampled. Then, using coordinates obtained in the field, the distance from each shrub to the ocean and to the closest forest patch were calculated using satellite images from Google Earth Pro 7.1.2.

### Statistical analysis

We used generalized linear models (GLMs) to compare abundance and richness of woody species and bromeliads in the two conditions (under the canopy of *G. opposita* individuals and in open areas). In each model, the explanatory variable ‘condition’ was considered a fixed effect. A Poisson distribution with a logarithmic function was used to relate response (species abundance and richness) and the explanatory variable.

To evaluate the strength of facilitation exerted by the shrub in relation to the adjacent area, the relative interaction index (RII) was used (Armas *et al.* 2004), as:

$$RII=\frac{B_{w}-B_{o}}{B_{w}+B_{o}}$$

were *B*_*w*_ is the number of individuals or species growing under the influence of *G. opposita* and *B*_*o*_ is the number of individuals or species growing outside the shrub influence (open areas). The resulting values can vary from −1 to 1, where positive values show facilitation and negative values mean competition (Armas *et al.* 2004).

We used GLMs to test how *G. opposita* facilitates plant abundance and species richness under the canopy. The explanatory variables used as fixed effects were height and canopy area, distance to the ocean, to the closest forest patch and to the closest freshwater body. Model selection was based on the Akaike information criterion (AIC). Statistical analysis were performed and figures were built using the packages ‘car’, ‘MASS’ and ‘ggplot2’ of the statistical software R version 2.3.1 (R Core Team 3.2.5).

## Results

The 35 sampled *G. opposita* individuals ranged in height between 0.8 and 3 m (mean 1.84 ± 0.11 m) and had a canopy area between 0.24 and 9.9 m^2^ (mean 3.74 ± 0.52 m^2^). These individuals were found 55 to 600 m from the ocean (mean 249.37 ± 21.68 m) and 1 to 25 m from a freshwater body (mean 5.74 ± 1.03 m). The distance to the closest forest patch varied from 95 to 710 m (372.51 ± 31.23 m). Relative interaction index values were positive both for abundance (0.28 ± 0.09) and richness (0.22 ± 0.08), showing a facilitation effect of *G. opposita* in this *restinga* environment.

Out of a total of 770 individuals, 537 (69.7 %) were recorded under *G. opposita* and 233 (30.3 %) in open areas. Of the 27 species recorded, 17 (62.9 %) occurred exclusively under the shrub canopy and 10 (37.1 %) were present in both conditions. Only four species (14.8 %) were more abundant in open areas but none of them were exclusively in this situation. Trees were the most frequent life form, followed by shrubs and subshrubs. Most of the species are typical of *restinga* with a shrubby vegetation physiognomy **[see****Supporting Information—Table S1****]**. Abundance and richness of woody plant and bromeliad species were higher under *G. opposita* canopies than in adjacent open areas (Table 1). Mean abundance and species richness under *G. opposita* canopies more than doubled that of open areas (Table 1).

**Table 1.** Abundance and richness of woody and bromeliad species, and results of the GLMs comparing both parameters under the canopies of *Guapira opposita* (canopy) and in adjacent open areas (open) of a coastal dune ecosystem, in Florianópolis, Santa Catarina, Brazil.

		Mean (±SE)	Min–Max	AIC	df	*Z*	*P*
Abundance	Canopy	15.89 ± 2.62	0–66	1060.2	68	−11.14	>0.01
	Open	6.66 ± 1.45	0–39				
Richness	Canopy	3.94 ± 0.48	0–10	301.16	68	−4.85	>0.01
	Open	1.91 ± 0.23	0–5				

**Figure 1.**
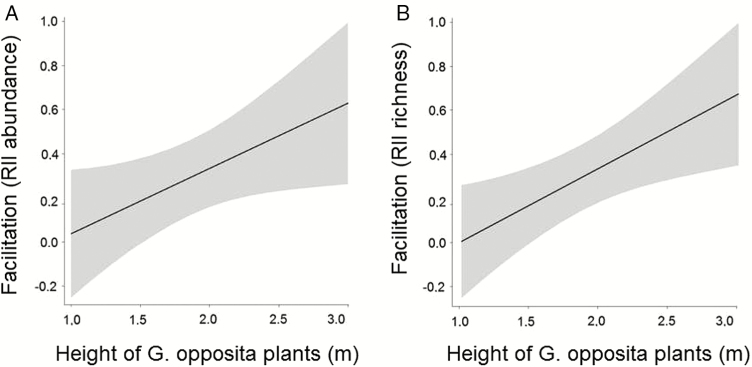
Regressions between RII and shrub height considering (A) abundance (*R*^2^ = 0.48, *P* = 0.03, *t* = 2.14) and (B) richness (species richness: *R*^2^ = 0.65, *P* = 0.01, *t* = 1.72) of woody species under the canopy of *Guapira opposita* shrubs in a coastal dune ecosystem (Florianópolis, Santa Catarina, Brazil). The areas in grey represent the confidence interval of each regression (±1.96 SEs).

With regards to RII values, models that best fit the data were those that included shrub height, distance to freshwater bodies and distance to forest patches (Table 2) **[see****Supporting Information—Table S2****]**. However, only shrub height was significant, explaining 48 % and 65 % of variation in abundance and richness, respectively (Fig. 1) **[see****Supporting Information—Table S2****]**. There was a positive relationship between shrub height and abundance of individuals and species richness (Fig. 1). The variables canopy area and distance to the ocean were not included in the best fit models.

**Table 2.** Models for abundance and richness of woody and bromeliad species under the canopy of *Guapira opposita* individuals sampled in a coastal dune ecosystem, in Florianópolis, Santa Catarina, Brazil. The model chosen (lowest AIC) for each parameter is in bold. dist. = distance.

	Fixed effects	AIC	ΔAIC	Pseudo-*R*^2^
Models for abundance				
Model 1	**height + dist. vegetation + dist. freshwater**	55.2	0.00	0.22
Model 2	height + dist. freshwater	55.7	0.05	0.17
Model 3	height + dist. vegetation + dist. freshwater + dist. ocean	57.1	1.90	0.22
Model 4	height	57.2	2.00	0.08
Model 5	dist. freshwater	57.2	2.00	0.07
Model 6	dist. vegetation	58.8	3.60	0.04
Models for richness				
Model 1	**height + dist. vegetation + dist. freshwater**	49.3	0.00	0.28
Model 2	height + dist. freshwater	49.6	0.30	0.23
Model 3	area + height + dist. vegetation + dist. freshwater + dist. ocean	51.4	2.10	0.14
Model 4	height	51.4	2.10	0.14
Model 5	dist. freshwater	51.7	2.40	0.05
Model 6	dist. vegetation	52.6	3.30	0.04

## Discussion

Our results show that both abundance and richness of woody species and bromeliads are higher under the canopy of *G. opposita* shrubs than in adjacent open areas. They also confirm that facilitation provided by this species is stronger under taller shrubs. Despite the fact that distance to freshwater bodies and distance to forest patches were not significant in the best fit model, the combined effect of these variables with shrub height best explained data variation, suggesting gradients not strong enough to influence interaction intensity. The canopy area of *G. opposita* plants and distance to the ocean were not relevant for facilitation under our shrub species.

Evidence of a positive interaction between nurse plants and their understory species has been widely reported in several ecosystems around the world, most notably in dry and high-elevation habitats (Baumeister and Callaway 2006; Zhao *et al.* 2007; Pugnaire *et al.* 2011; Schöb *et al.* 2012; Semchenko *et al.* 2012; Cavieres *et al.* 2014). Reports of facilitation in tropical and subtropical ecosystems are far less frequent. There are, however, reports from tropical grasslands (Tirado and Pugnaire 2005), high mountains (Anthelme *et al.* 2015) and *restinga* environments (Castanho *et al.* 2012). Such a low availability of published data concerning the tropics may be related to the formulation of the SGH, which led to search for positive interactions in stressful environments (Bertness and Callaway 1994). However, in subtropical coastal systems there may be significant levels of plant stress related to nutrient limitation, low soil water availability and/or high salinity levels (Hesp 1991) which would make facilitation important for plant community dynamics.

In our coastal system, despite abundant rainfall and high mean annual temperature, different stress factors lead to a rather demanding environment, such as high radiation, high salinity levels, desiccating winds and low nutrient availability (Castanho *et al.* 2015). Therefore, the higher plant abundance and species richness recorded under the canopy of *G. opposita* can be explained mainly by shading which, along with lower soil temperatures, organic matter and nutrient accruement, favoured plant establishment and niche creation (Moro *et al.* 1997; Bruno *et al.* 2003; Schöb *et al.* 2012). Shade also keeps temperatures close to the plant physiological equilibrium, as temperatures above 50–60 °C causes cellular damage (Callaway 2007), reducing evaporation and keeping better plant water balance (Pugnaire *et al.* 2011). Some of these changes may affect interaction intensity by, e.g., increasing competition among beneficiary species (Schöb *et al.* 2014), but the overall effect of the canopy would allow the presence of more individuals and increased species richness under the canopy of the facilitator species (Moro *et al.* 1997; Castanho *et al.* 2012; McIntire and Fajardo 2014).

Facilitation provided by *G. opposita* shrubs is higher when shrubs are taller, presumably by two factors—the darker shade they cast and the perch effect. Taller plants are more attractive to seed dispersers (Debussche *et al.* 1982) and seed rain is larger under perches (Pausas *et al.* 2006), while nurse shadow favours seed germination and seedling establishment. The most abundant woody species found under the canopy of *G. opposita* have fleshy fruits dispersed by birds—*C. criuva*, *G. brasiliensis*, *M. palustris*, *O. pulchella* and *G. opposita* as predicted as consequences of the perch effect (Arantes *et al.* 2014). Species in the Lauraceae, Myrtaceae and Primulaceae were found exclusively under *G. opposita***[see****Supporting Information—Table S1****]**, in accordance with previous reports where these species were described as typical of arboreal *restinga* physiognomy and rarely found in herbaceous *restinga* environments (Guimarães 2006). This shows that *G. opposita* facilitates preferentially the establishment of tree species that otherwise do not have the capacity to recruit in exposed *restinga* sites.

In addition, most species found under *G. opposita* are typical in arboreal *restingas* (Guimarães 2006) and were more abundant in forest patches than in the herbaceous matrix. This is an evidence that, although distance to forest patches was not significant in the best fitted model, it contributes to the observed results. In the studied system, forest patches act as seed sources and isolated shrubs (such as *G. opposita* plants) function as stepping stones for seed dispersers in an herbaceous matrix.

Some 25 % of the individuals sampled under the canopy of *G*. *opposita* were not found outside this influence **[see****Supporting Information—Table S1****]**, which shows the critical contribution of this shrub species to local biodiversity. For instance, the bromeliad *Vriesea fribugensis* is a wind-dispersed species that thrives in environments with diffuse light and which allow water to accumulate in its leaves (Reitz 1983). By contrast, two of the three most common species found in open areas (*Baccharis dracunculifolia* and *Dodonaea viscosa*) are anemochorous shrubs and tolerate environments of extreme radiation (Guimarães 2006).

Distance to freshwater bodies may be a limiting factor in *restinga* environments as soil humidity can limit the development of plants (Wilson and Sykes 1999). Therefore, it could be expected a facilitation gradient since water availability decreases with increasing distance to freshwater bodies, in agreement with the SGH (Bertness and Callaway 1994). However, there was not a direct relationship between distance to freshwater bodies and facilitation under *G. opposita*, which implies that soil humidity is not a limiting resource for the studied system. In fact, short distances to freshwater bodies could also be considered as a stressor for the local plant communities due to (un)predictable flooding (Scarano *et al.* 1997). These mixed effects of distance to freshwater bodies must have neutralized its relevance on the best fit model.

Salinity, here represented by distance to the ocean as a proxy, is also a stress factor in coastal environments (Shumway 2000). Contrary to our expectations, it did not determine abundance of individuals under *G. opposita* canopies (Castanho *et al.* 2012), as the 600 m distance to the ocean seems not to be strong enough to notice the effects of salinity on plant establishment.

## Conclusions

We conclude that *G. opposita* shrubs increased richness and abundance of other woody and bromeliad species under its canopy compared to the surrounding herbaceous matrix. The main factor determining facilitation intensity was individual shrub size (shade and perch effect) which positively influenced native species abundance and richness. Also, our data show that shrub canopy area, distance to forest patches, distance to freshwater bodies and distance to the ocean (as a proxy for salinity) were not important factors influencing facilitation. To sum up, subtropical environments may be stressful for plants and nurse species play a key role in the regeneration of *restinga* environments, where their presence is critical to maintain ecosystem diversity and function.

## Sources of Funding

This work was funded by the Brazilian government (CAPES/CNPq). C.E.S.D. and R.B.S. were recipient of scholarships from the Ecology Postgraduate Program of the Federal University of Santa Catarina (UFSC). F.I.P. and M.S.D. were supported by the CAPES/CNPq ‘Science without Borders’ Program, through the project ‘Plant interactions and community dynamics in tropical, seasonal systems’ (ref. UFSC 114A-2013).

## Contributions by the Authors

C.E.S.D., F.I.P. and T.T.C. conceived the original ideas. C.E.S.D., R.B.S. and M.S.D. executed the fieldwork and analysed the data. C.E.S.D. wrote the first draft of the manuscript with contributions of all co-authors.

## Conflict of Interest

None declared.

## Supporting Information

The following additional information is available in the online version of this article—

**Table S1.** Total abundance of woody and bromeliad species sampled under the canopies of *Guapira opposita* (canopy) and adjacent open areas (open) in a coastal dune ecosystem, in Florianópolis, Santa Catarina, Brazil. Life form and occurrence in each of the *restinga* physiognomies are showed—herbaceous (H), shrubby (S) and arboreal (A).

**Table S2.** Statistical results of the chosen generalized linear models (GLMs) built to explain the relative interaction index (RII) for abundance and richness of woody and bromeliad species sampled under the canopies of *Guapira opposita* (canopy) and adjacent open areas (open) in a coastal dune system, in Florianópolis, Santa Catarina, Brazil. df = degrees of freedom; dist = distance.

Supporting Information
