# Supplementary material for: Facilitation influences patterns of perennial species abundance and richness in a subtropical dune system
Source: AoB Plants. 2018 Mar 23;10(2):ply017. doi: 10.1093/aobpla/ply017 (PMC5888537; doi:10.1093/aobpla/ply017)
Supplement: Supporting Information [file ply017_suppl_supporting_information.docx]

**Table S1** – Total abundance of woody and bromeliad species sampled under the canopies of *Guapira opposita* (canopy) and adjacent open areas (open) in a coastal dune ecosystem, in Florianópolis, SC, Brazil. Life form and occurrence in each of the *restinga* physiognomies are showed - herbaceous (H), shrubby (S) and arboreal (A).

| **Botanical family** | **Species** | **Canopy** | **Open** | **Life form** | **Physiognomy** |
| --- | --- | --- | --- | --- | --- |
| **Anacardiaceae** | *Lithraea brasiliensis* | 20 | 0 | Tree | S |
|  | *Schinus terebinthifolius* | 3 | 0 | Tree | H,S |
| **Asteraceae** | *Baccharis dracunculifolia* | 7 | 79 | Shrub | S |
|  | *Porophyllum ruderale* | 3 | 2 | Subshrub | H |
| **Bromeliaceae** | *Aechmea lindenii* | 12 | 0 | Herb | S,A |
|  | *Vriesea friburgensis* | 161 | 18 | Herb | H,S |
| **Clusiaceae** | *Clusia criuva* | 46 | 5 | Tree | A |
| **Ericaceae** | *Gaylussacia brasiliensis* | 33 | 6 | Shrub | H,S |
| **Euphorbiaceae** | *Alchornea triplinervia* | 3 | 0 | Tree | A |
| **Fabaceae** | *Stylosanthes viscosa* | 2 | 20 | Subshrub | H,S |
| **Lamiaceae** | *Vitex megapotamica* | 16 | 51 | Shrub | H,S |
| **Lauraceae** | Lauraceae sp1. | 2 | 0 | - | - |
|  | Lauraceae sp2. | 1 | 0 | - | - |
|  | Lauraceae sp3. | 1 | 0 | - | - |
|  | Lauraceae sp4. | 1 | 0 | - | - |
|  | *Ocotea pulchella* | 29 | 1 | Tree | S,A |
| **Myrtaceae** | *Eugenia catharinae* | 16 | 0 | Tree | S,A |
|  | *Myrcia* cf*. multiflora* | 11 | 0 | Tree | A |
|  | *Myrcia palustris* | 33 | 0 | Tree | S |
| **Nyctaginaceae** | *Guapira opposita* | 69 | 8 | Tree | S,A |
| **Primulaceae** | *Myrsine parvifolia* | 3 | 0 | Tree | S |
|  | *Myrsine umbellata* | 11 | 0 | Tree | A |
|  | *Myrsine venosa* | 26 | 0 | Tree | A |
| **Rubiaceae** | *Posoqueria latifolia* | 1 | 0 | Tree | A |
|  | *Chiococca alba* | 1 | 0 | Shrub | S |
|  | *Psychotria carthagenensis* | 3 | 0 | Shrub | A |
| **Sapindaceae** | *Dodonaea viscosa* | 23 | 43 | Shrub | H,S |

**Table S2 –** Statistical results of the chosen generalized linear models (GLM) built to explain the Relative Interaction Index (RII) for abundance and richness of woody and bromeliad species sampled under the canopies of *Guapira opposita* (canopy) and adjacent open areas (open) in a coastal dune system, in Florianópolis, SC, Brazil. df = degree of freedom. dist = distance.

| **Model** | **Variables** | **t** | **p** |
| --- | --- | --- | --- |
| **Abundance** | height | 2.14 | 0.03 |
| AIC = 55.2/ df = 31 | dist. vegetation | -1.53 | 0.13 |
|  | dist. freshwater | 1.62 | 0.11 |
|  |  |  |  |
| **Richness** | height | 2.74 | <0.01 |
| AIC = 49.31/ df=31 | dist. vegetation | -1.44 | 0.15 |
|  | dist. freshwater | 1.72 | 0.09 |
